# Expression of *FoxA *and *GATA *transcription factors correlates with regionalized gut development in two lophotrochozoan marine worms: *Chaetopterus *(Annelida) and *Themiste lageniformis *(Sipuncula)

**DOI:** 10.1186/2041-9139-1-2

**Published:** 2010-07-05

**Authors:** Michael J Boyle, Elaine C Seaver

**Affiliations:** 1 Kewalo Marine Laboratory, Pacific Biosciences Research Center, University of Hawaii, Honolulu, HI 96813, USA

## Abstract

**Background:**

A through gut is present in almost all metazoans, and most likely represents an ancient innovation that enabled bilaterian animals to exploit a wide range of habitats. Molecular developmental studies indicate that *Fox *and *GATA *regulatory genes specify tissue regions along the gut tube in a broad diversity of taxa, although little is known about gut regionalization within the Lophotrochozoa. In this study, we isolated *FoxA *and *GATA456 *orthologs and used whole mount *in situ *hybridization during larval gut formation in two marine worms: the segmented, polychaete annelid *Chaetopterus*, which develops a planktotrophic larva with a tripartite gut, and the non-segmented sipunculan *Themiste lageniformis*, which develops a lecithotrophic larva with a U-shaped gut.

**Results:**

*FoxA *and *GATA456 *transcripts are predominantly restricted to gut tissue, and together show regional expression spanning most of the alimentary canal in each of these lophotrochozoans, although neither *FoxA *nor *GATA456 *is expressed in the posterior intestine of *Chaetopterus*. In both species, *FoxA *is expressed at the blastula stage, transiently in presumptive endoderm before formation of a definitive gut tube, and throughout early larval development in discrete foregut and hindgut domains. *GATA456 *genes are expressed during endoderm formation, and in endoderm and mesoderm associated with the midgut in each species. Several species-specific differences were detected, including an overlap of *FoxA *and *GATA456 *expression in the intestinal system of *Themiste*, which is instead complimentary in *Chaetopterus*. Other differences include additional discrete expression domains of *FoxA *in ectodermal trunk cells in *Themiste *but not *Chaetopterus*, and expression of *GATA456 *in anterior ectoderm and midgut cells unique to *Chaetopterus*.

**Conclusions:**

This study of gene expression in a sipunculan contributes new comparative developmental insights from lophotrochozoans, and shows that *FoxA *and *GATA456 *transcription factors are part of an ancient patterning mechanism that was deployed during early evolution of the metazoan through gut. The common utilization of *FoxA *and *GATA456 *throughout gut formation by species with contrasting life history modes indicates that both genes are core components of a gut-specific gene regulatory network in spiralians. Despite a highly conserved pattern of early development, and probably similar ontogenic origins of gut tissue, there are molecular differences in gut regionalization between lophotrochozoan species.

## Background

Formation of a through gut was a key innovation during the radiation of the Metazoa. Although the origins of an internal digestive tract are uncertain, it probably occurred early in animal evolution [1,2]. Considering this, molecular mechanisms that specify cells and tissues of such an ancient organ system are expected to show some level of conservation across a broad range of taxa [1,3,4]. Multiple studies support this expectation, especially for several transcriptional regulators of endoderm [3,5,6]^7^,^8^. Over evolutionary time, animals generated a variety of digestive organs and adaptive feeding mechanisms that enabled them to utilize a wide range of food sources [9]. How different gut anatomies develop, how planktotrophic and lecithotrophic life histories influence gut evolution, whether larval and adult intestinal systems are fundamentally different, and which molecular mechanisms explain the variation in gut morphology between closely related animals are still not well understood. In a previous study [10], we described morphologic and molecular aspects of gut development in the marine polychaete, *Capitella teleta *Blake, Grassle & Eckelbarger 2009, known for years as *Capitella *sp. I. In this paper, we extend that work with developmental data obtained from two additional marine worms.

The 'segmented' annelid worms are one of the most speciose and ecologically diversified protostome animal groups, and are well represented within one of three metazoan superclades, the understudied Lophotrochozoa [11]. One of our study species is the parchment worm, *Chaetopterus variopedatus *Cuvier 1827, sensu Enders (1909), which M. E. Petersen (personal communication) suggested is most likely not *C. variopedatus*, but an undescribed species. We refer to this polychaete as *Chaetopterus*, a marine annelid that has been used both in classic [12-14] and more recent [15-18] developmental studies. *Chaetopterus *lives in U-shaped tubes within sublitorral, mud-bottom habitats along the Northeast Atlantic coastline and presumably is a broadcast spawner [19]. Recent phylogenetic analyses consider the Chaetopteridae as one of the more basal annelid groups [20].

The second species is a sipunculan worm, *Themiste lageniformis *Baird 1868, for which there are a few studies describing its reproduction [21,22] and general patterns of development [21,23]. Adult females of *T. lageniformis *are considered to reproduce by facultative parthenogenesis, and both sexes are distributed in benthic marine habitats along coastlines of Africa, India, Indo-Pacific, Hawaii and Florida [21,24]. The occurrence of fossil sipunculans from the Lower Cambrian implies they are an ancient group of animals [25], and may be the sister group to annelids [26]. However, an accumulation of data from morphologic [27-30], mitochondrial [31,32] and molecular phylogenetic sources [20,33-35] suggest that the monophyletic Sipuncula [36] belong within Annelida, although they are not consistently affiliated with any particular annelid group.

These two species provide a valuable comparison for examining the evolutionary development of gut formation because *Chaetopterus *and *T. lageniformis *develop very different larval and adult gut morphologies. Descriptions of larval morphology [37], anatomy [38] and development [15,39,40] demonstrate that *Chaetopterus *larvae form a functional, tripartite intestinal system consisting of the pharynx, midgut and hindgut. The larvae of *T. lageniformis *form a U-shaped gut without distinct compartments [23]. Moreover, their respective life histories are dissimilar. *Chaetopterus *larvae are planktotrophic, begin feeding within 36 hours of development, and they are capable of extended planktonic periods exceeding 30 days [40]. The unique pelagosphera larvae of *T. lageniformis *are lecithotrophic, swimming on or near the bottom in laboratory cultures for up to 9 days [41], and do not feed until after metamorphosis. In both species, metamorphosis includes substantial changes in gut morphology between larval and juvenile forms.

We are interested in understanding the molecular basis for distinct regional differences in gut morphology. Important candidate genes likely to be involved in gut regionalization include both *Fox *and *GATA *factors. The *FoxA *('Forkhead box' A) gene belongs to a multi-family group of 'winged-helix' transcription factors with structurally similar 110-amino acid DNA-binding domains [42,43]. *GATA *genes belong to a family of transcription factors with one or two class IV zinc-finger motifs [44]. Individual GATA factors are typically assigned to one of two subclasses, GATA1/2/3 or GATA4/5/6 [44], and their evolution has been described in detail [45]. Both *FoxA *and *GATA456 *gene members are part of a larger endomesoderm specification network of genes that have been shown to have regulatory roles in endoderm and mesoderm formation during embryogenesis and larval morphogenesis in a nematode [46], fly [47], sea urchin [48-50] and frog [51], and are expressed in similar tissues in many other animals. Within Annelida, gene expression data suggest that two paralogs of *FoxA *are in gut precursor cells of *Hydroides elegans *[52], and there is mesodermal, although no endoderm expression, of a *GATA456 *ortholog in *Platynereis dumerilii *[53]. Only for the polychaete *C. teleta *are there expression data for transcription factors from both gene families, and their patterns are clearly implicated in gut formation and regionalization [10].

In this study, we isolated and identified *FoxA *and *GATA456 *orthologs from *Chaetopterus *and *T. lageniformis*. We then characterized their expression patterns to help clarify whether these genes in annelids have the potential to act as conserved or novel key regulators of the gut developmental process [50,54,55]. We also describe gut formation for both species to guide interpretation of *in situ *hybridization patterns. In each worm, *FoxA *and *GATA456 *factors show a pattern of regionalized expression in both ectodermal and endodermal domains of the developing gut tube. We interpret these expression patterns in the context of life history mode and the inherent morphogenesis associated with gut formation. This is the first report of any gene expression from a member of the Sipuncula.

## Results

### Larval gut formation in *Chaetopterus*

**Figure 1:**
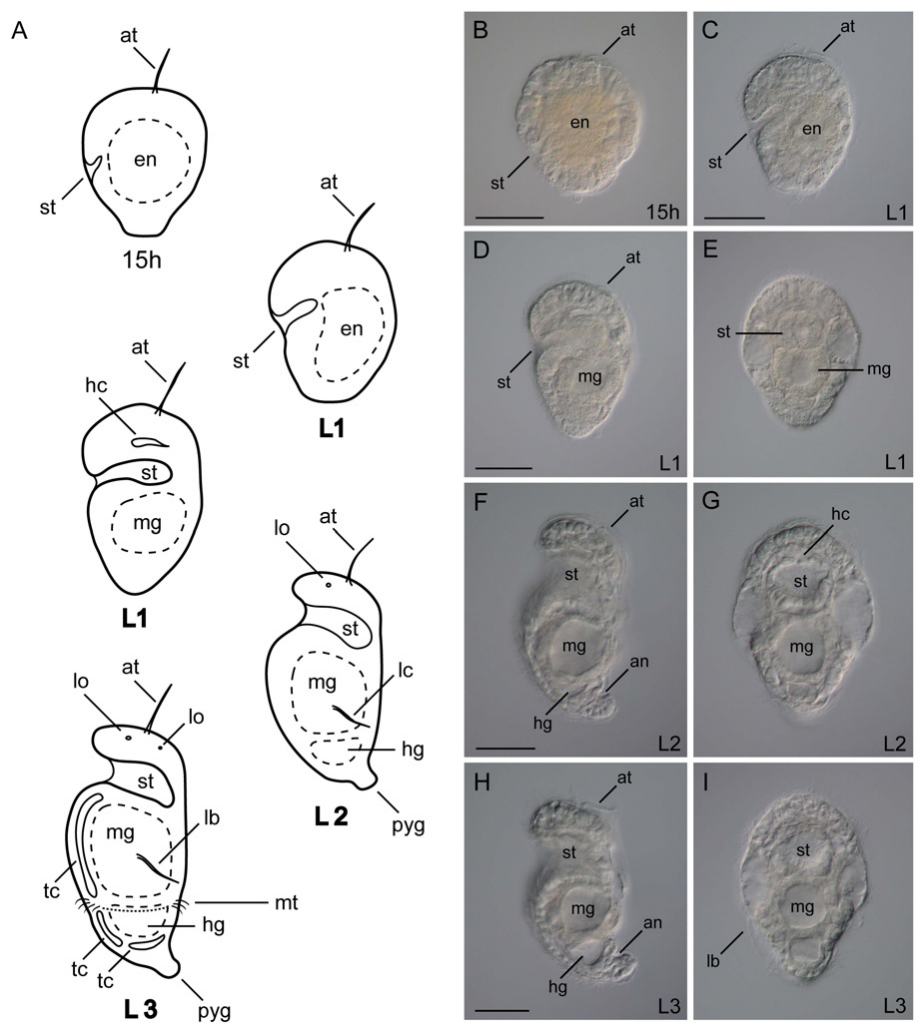
**Early stages of larval gut development in *Chaetopterus***. (A) Lateral view schematics with anterior to the top and ventral to the left. Gut formation and regionalization in larval stages at 15 hours, L1 (18 to 36 hours), L2 (36 to 72 hours) and L3 (3 to 30 days). (B-I) DIC micrographs of larval stages corresponding to schematics in (A). (B, C, D, F, H) Lateral views with anterior to the top and ventral left; (E, G, I) ventral views with anterior to the top. an, anus; at, apical tuft; en, endoderm; hc, head coelom; hg, hindgut; lb, lateral bristle; lc, lateral hooked cilia; lo, larval ocellus; mg, midgut; mt, mesotroch; pyg, pygidium; st, stomodeum; tc, trunk coelom. Scale bars = 50 μm. Larval schematics modified from Irvine *et al. *[40].

The anatomy and morphology of post-gastrulation larval development in *Chaetopterus *has been documented [40]. Here, we describe the general features of gut formation during the early stages of embryonic and larval growth, and add to the published staging system to emphasize gut characteristics pertinent to this study (Figure 1). After gastrulation, the stomodeum is detectable approximately 11 hours post-fertilization, and is formed as an invagination on the posterior ventral face of the embryo. During the developmental period, from 15 hours through the early L1 stage, the stomodeum elongates in an anterior-dorsal direction through the larva's interior (Figure 1B, C). At the same time, the region of yolk-rich endoderm cells is shifted posteriorly (Figure 1C), where they will form the midgut. In the late L1, the dorsal-most end of the stomodeum is directly anterior and adjacent to a midgut epithelium, which is now circular and encloses the midgut cavity (Figure 1D, E). The hindgut has a ciliated canal (not shown) but no obvious epithelium or lumen is visible with differential interference contrast (DIC) optics at this stage. Both the stomodeum and midgut lumens are lined with cilia in late L1 larvae. In L2 larvae, the gut is functional [40]. At its opening on the ventral face, the stomodeum extends laterally across most of the width of the larva, and its ciliated canal narrows as it extends inward (Figure 1A, F, G). There is a valve on the floor of the stomodeal canal at the foregut-midgut junction [40]. The L2 midgut is lined with relatively long cilia, and its lumen forms a large conspicuous cavity in the larva's midbody (Figure 1F, G). The hindgut is positioned directly posterior to the midgut, and its lumen becomes visible and lined with cilia at L2 (Figure 1F, G). The midgut and hindgut are connected by a canal that passes through the ventral posterior epithelium of the midgut. A rectal canal connects the ventral posterior hindgut cavity to the anus, and exits the larva through the dorsal epidermis, anterior to the pygidium (Figure 1F). The entire length of the gut is ciliated. All of the above features are present and more pronounced in the L3 larva of *Chaetopterus *(Figure 1A, H, I).

### Embryonic and larval gut formation in *Themiste lageniformis*

**Figure 2:**
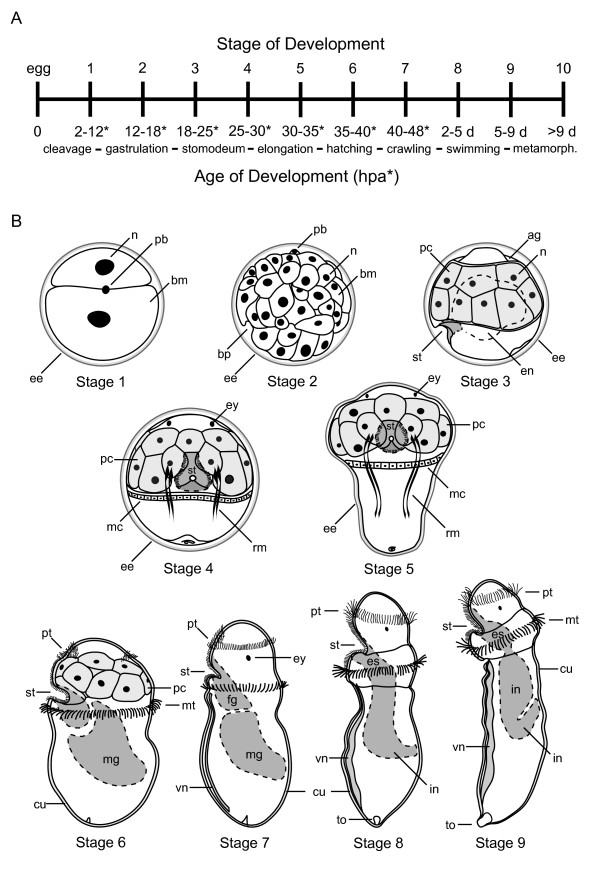
**Schematic of embryonic and larval development in the sipunculan, *Themiste lageniformis***. (A) Timeline showing stages of development and corresponding age intervals in hours post-activation (HPA), along with a series of major developmental events that characterize the transition to each stage. (B) Diagrams of developmental stages 1 to 9, which depict prominent morphologic-characters occurring from first cleavage through formation of the non-feeding, pre-metamorphic pelagosphera larva. Stage 1, animal-pole view; stage 2, lateral view with animal hemisphere up; stages 3, 6, 7, 8 and 9 are lateral views with ventral to the left and anterior up; stages 4 and 5 are ventral views with anterior up. ag, apical groove; bm, blastomere; bp, blastopore; cu, cuticle; ee, egg envelope; en, endoderm; es, esophagus; ey, eye; fg, foregut; in, intestine; mc, metatroch cells; mg, midgut; mt, metatroch; n, nucleus; pb, polar body; pc, prototroch cell; pt, prototroch; rm, retractor muscle; st, stomodeum; to, terminal organ; vn, ventral nerve cord. Light gray shading in stages 3 to 6, prototroch cells; dark gray shading in stages 3 to 9, gut tissue; ventral nerve cords also are shaded gray in stages 7 to 9.

**Figure 3:**
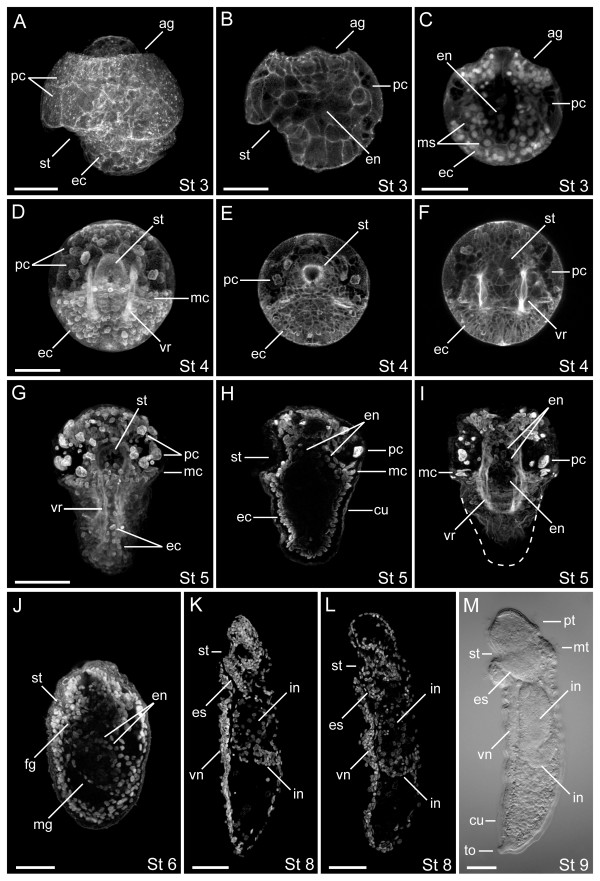
**Micrographic analysis of gut formation in the sipunculan, *Themiste lageniformis***. (A-L) confocal laser scanning micrographs (CLSM). Actin filaments are labeled with BODIPY phallacidin (A-I); cell nuclei are labeled with anti-histone antibody (C-J) or propidium iodide (K, L); (M), light micrograph with DIC optics. (A, B, H, J-M) lateral views with ventral to the left and anterior up; (C, I) dorsal views with anterior up; (D-G) ventral views with anterior up. (A) Three dimensional z-series of a stage 3 embryo showing position of the stomodeum. (B) Single focal plane of the embryo in (A). (C) Dorsal view z-series of a stage 3 embryo showing relative positions of ectoderm, mesoderm and endoderm cells. (D) Three dimensional z-series of a stage 4 embryo before elongation. The stomodeum is closed at its dorsal end. (E, F) Two z-series composites of the same embryo in (D), at progressively deeper focal planes. The stomodeum forms a 'bowl' that is lined with ciliated cells, and terminates with a rosette of larger cells. (G-I) Ventral, lateral and dorsal views of three different conical-shaped stage 5 embryos. The stomodeum is oriented parallel to the D/V axis just anterior to the metatroch. (J) Stage 6 larva with the stomodeum in an anterior to posterior orientation, and midgut with endoderm nuclei. (K, L) Early and late stage 8 pelagosphera, respectively. The esophagus is packed with cells and the intestine ascends toward the dorsal body wall. (M) Stage 9 pelagosphera after 4 days of growth. Descending and ascending arms of the intestinal system are now visible. ag, apical groove; cu, cuticle; ec, ectoderm cells; en, endoderm cells; fg, foregut; hg, hindgut; mc, metatroch cells; mg, midgut; ms, mesoderm cells; mt, metatroch; pc, prototroch cells; pt, prototroch; st, stomodeum; to, terminal organ; vn, ventral nerve cord; vr, ventral retractor muscle. Scale bars = 50 μm.

General characteristics of reproduction, development and metamorphosis in *Themiste lageniformis *were previously described as part of a doctoral dissertation [23]. Here, we describe both embryonic and larval aspects of gut formation during pre-metamorphic development in *T. lageniformis*. To guide our interpretations of gene expression for this species, we have generated a developmental staging system representing early cleavage through larval development (Figure 2A) that includes diagrammatic views and descriptions of prominent stage-specific features (Figure 2B). Additionally, we provide detailed confocal micrographs emphasizing gut morphogenesis for selected stages (Figure 3). The pelagosphera larva of *T. lageniformis *is lecithotrophic and non-feeding, and therefore the gut is non-functional until the juvenile stages.

Approximately 2 hours after chemically activating (see Methods) mature oocytes of *T. lageniformis*, a unipolar cleavage furrow moves across the egg surface, initiating a conserved program of spiral, holoblastic cleavage. At the completion of first cleavage, a single polar body is visible on the animal pole and the egg is divided into a large CD cell and a smaller AB cell (Figure 2B). The D cell is the largest blastomere at the four-cell stage, and at eight cells, micromeres of the A, B and C quadrants are larger than their respective macromeres, with the D macromere being the largest of all eight blastomeres (not shown). In mid blastula (stage 1), a single polar body identifies the animal pole, and relatively large, yolky cells are positioned at the vegetal pole (Figure 2B). During late stage 2, gastrulation occurs by epiboly, and a blastopore forms on the posterior ventral face of the embryo [23]. The stomodeum is detectable at stage 3 as a pronounced indentation on the embryo's ventral posterior face, and initially extends in a dorsal-anterior direction under the posterior rim of a band of conspicuously large, non-ciliated prototroch cells (Figure 2B, Figure 3A-C). The pretrochal anterior end of the stage 3 embryo is marked by an apical groove [56] between the animal pole and the prototroch. Internally, there is a centralized yolk-rich region visible in both live and fixed specimens (Figure 2B, Figure 3C), and presumptive mesoderm cells flank each side of the endoderm (Figure 3C). Stage 4 is characterized by an episphere with a pair of larval eyes, a band of large prototroch cells, bilateral sets of retractor muscle fibers on each side of the endoderm, and a circumferential band of non-ciliated metatrochal cells directly posterior to the prototroch (Figure 2B, Figure 3D-F). The stomodeum is rimmed with ciliated cells, and forms a 'bowl-shaped' cavity that interrupts the ventral-most pair of prototroch cells (Figure 2B, Figure 3D). The stomodeum is closed at its dorsal end, where it is lined with a rosette of larger cells (Figure 3F). Endoderm cells are situated in the yolky interior, posterior to the stomodeum, although they are difficult to visualize at this stage. Between stages 4 and 5, the hyposphere extends in a posterior direction, producing a conical-shaped embryo. The stomodeum is broader and extends deeper interiorly; however, it is still closed at its dorsal end where it abuts a group of interior cells (Figure 2B, Figure 3G-I). The ventral pair of head retractor muscles wrap around the stomodeal cavity at their anterior end (Figure 2G). Presumptive endoderm cell nuclei are located to the interior of the dorsal wall of the stomodeum, but are not detected in the central trunk region posterior to the metatrochal band (Figure 3H, I).

Stage 6 represents a transition to the pelagosphera larva, and is considered analogous to the first of two 'metamorphic' stages in many sipunculans [56]. The large prototroch cells are still present but reduced in size, and the metatroch is now ciliated. Repeated extensions and contractions of the body gradually cast off part of the egg envelope on the anterior end, and a larval cuticle is formed around the body [23]. At this stage, the stomodeum is open to the environment and lined with cilia at the surface. The interior of the stomodeum is packed with cells and now is oriented along the anterior-posterior (A/P) axis (Figure 2B, Figure 3J). The midgut contains numerous endoderm nuclei, and extends from the stomodeum through the trunk to the dorsal posterior side of the larva, narrowing along the A/P axis (Figure 3J). At stage 7, the large prototroch cells are reduced or lost, and ciliated cells surround the mouth and form a new prototrochal band that encircles the head. The metatroch is active, and propels the larva during crawling or gliding motions along the substrate surface. The ventral nerve cord is apparent as a condensation of nuclei along the ventral midline of the trunk. The foregut is packed with cells that will form the esophagus, and the midgut sweeps from ventral anterior to dorsal posterior (Figure 2B). Stage 8 larvae swim into the water column. These larvae now have an elongate esophagus region that extends posteriorly from the mouth to the intestine, which broadens at its posterior end and then narrows as it curves toward the larva's dorsal-posterior body wall (Figure 2B, Figure 3K, L). Williams [23] described both yolk granules and lipid droplets within membrane-bound gut cells, and we see yolky endoderm cells and cell nuclei (Figure 3K, L) dispersed along the length of the intestine. It is also clear from viewing live specimens that there is a considerable amount of yolk moving freely within the coelom. The pelagosphera larva at stage 9 has numerous cilia surrounding the mouth and ventral face of the head, also present in stages 7 to 8, and an actively beating metatrochal band (Figure 2B, Figure 3M). A thick cuticle covers the body, and at the posterior end there is a terminal organ, which serves the larva as an adhesive mechanism for attachment to the substrate [56]. The relatively compact esophagus is oriented in an anterior-posterior direction, and is constricted at its junction with the intestine (Figure 3M). The intestine is broader along its anterior section, narrows at the posterior end of the descending arm, and then curves and ascends toward its terminus where the rectum and anus will develop (Figure 2B, Figure 3M). Larvae at stages 8 and 9 are capable of fully retracting their anterior ends, which temporarily repositions most of the gut tube within the posterior end of the coelomic cavity.

### Characterization and orthology assignment of *FoxA *and *GATA *genes

**Figure 4:**
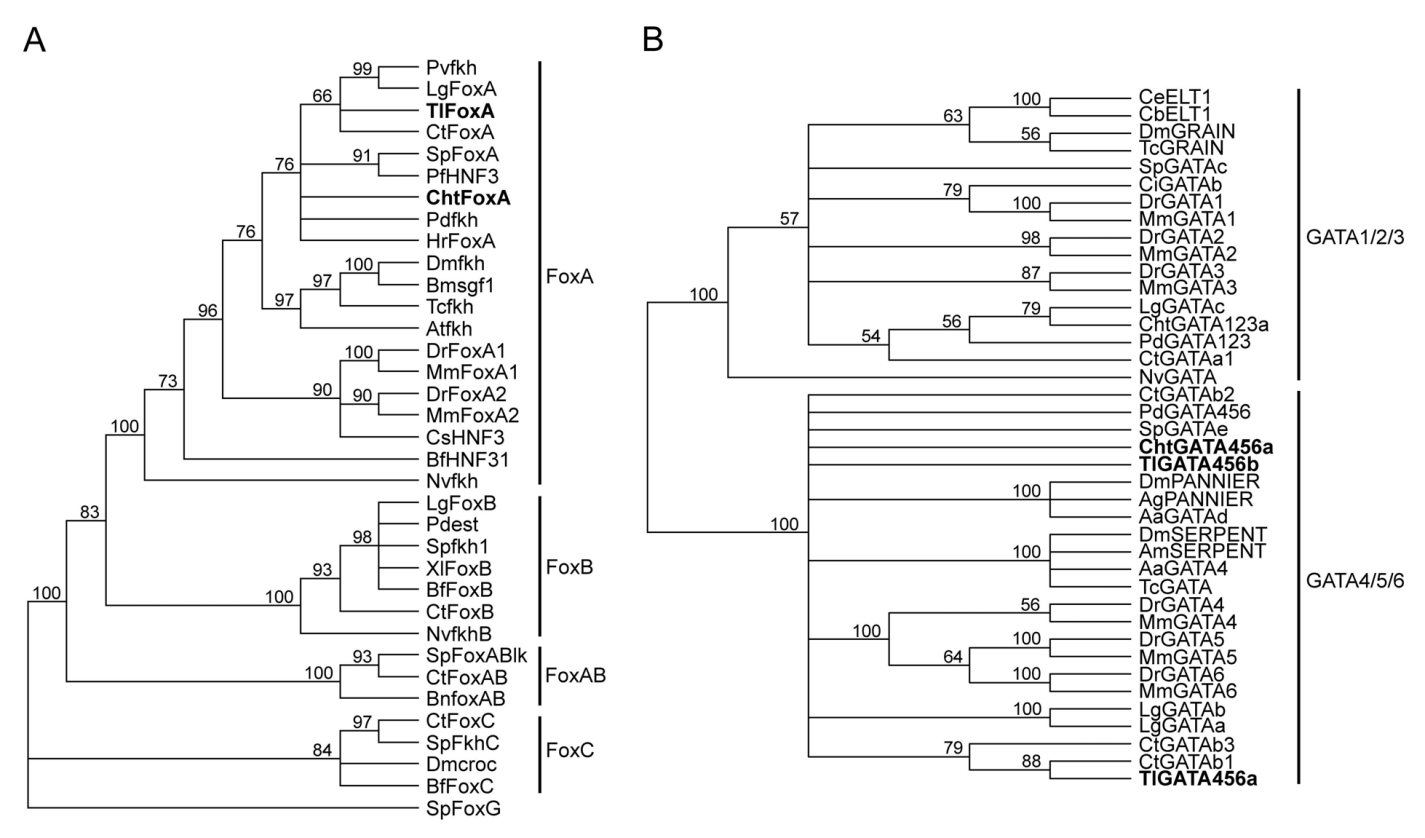
**Orthology analyses of FoxA and GATA4/5/6 DNA-binding proteins from *Chaetopterus *and *Themiste lageniformis***. (A) Unrooted Bayesian cladogram produced from the 110-amino acid, winged-helix domains of selected Fox family transcription factor genes. The topology is a 50% majority-rule consensus tree generated from 90,004 trees. (B) Unrooted Bayesian cladogram produced from the C_4 _dual zinc-finger domains in two prominent subclasses of GATA transcription factors. This topology is a 50% majority-rule consensus tree generated from 80,004 trees. Posterior probabilities are located at the nodes within each tree. Species abbreviations: Aa, *Aedes aegypti*; Ag, *Anopheles gambiae*; Am, *Apis mellifera*; At, *Achaearanea tepidariorum*; Bf, *Branchiostoma floridae*; Bm, *Bombyx mori*; Bn, *Bugula neritina*; Cb, *Caenorhabditis briggsae*; Ce, *Caenorhabditis elegans*; Cht, *Chaetopterus*; Ci, *Ciona intestinalis*; Cs, *Ciona savigny*; Ct, *Capitella teleta*; Dm, *Drosophila melanogaster*; Dr, *Danio rerio*; Hr, *Helobdella robusta*; Lg, *Lottia gigantea*; Mm, *Mus musculus*; Nv, *Nematostella vectensis*; Pd, *Platynereis dumerilii*; Pf, *Ptychodera flava*; Pv, *Patella vulgata*; Sp, *Strongylocentrotus purpuratus*; Tc, *Tribolium castaneum*; Tl, *Themiste lageniformis*

cDNA fragments of *Fox *and *GATA *transcription factor genes were isolated from the polychaete, *Chaetopterus*, and the sipunculan, *T. lageniformis*. Sequence analyses for all recovered *Fox *factors confirmed the presence of a characteristic 110-amino acid winged-helix domain. An amino acid alignment containing the winged-helix domains from both worms, along with similar domains in *FoxA *genes from additional metazoan taxa, was analyzed with Bayesian statistics and supports the identity of *FoxA *family members from both species (Figure 4A). The *GATA *factors were each observed to have a dual C_4 _zinc-finger domain, and a similar statistical analysis of these zinc-finger domains confirmed our recovery of *GATA *factors in the GATA4/5/6 subclass from each species (Figure 4B). Posterior probability (PP) values for internal nodes within each unrooted cladogram were moderately supportive for gene-specific associations between taxa; however there was 100% PP for a distinct FoxA family and two GATA subclasses, which strongly supports the respective orthology assignments for the genes we examine in this study. Although not described in detail here, a single gene from both worm species was also isolated and identified as a member of the GATA1/2/3 subclass, and we included *Cht-GATA123a *in this analysis to help resolve the two distinct GATA subclasses (Figure 4B). The *Chaetopterus FoxA *gene, *Cht-FoxA*, has a predicted open reading frame (ORF) of 1215 bp, and the *T. lageniformis FoxA *gene, *Tl-FoxA*, has a predicted ORF of 1416 bp. The *GATA *transcription factor gene isolated from *Chaetopterus*, *Cht-GATA456a*, was determined to have an ORF of 1794 bp, and the two *T. lageniformis GATA *factor genes, *Tl-GATA456a *and *Tl-GATA456b*, contain ORFs of 2193 bp and 1491 bp, respectively.

### Developmental expression of *Cht-FoxA *and *Cht-GATA456a*

**Figure 5:**
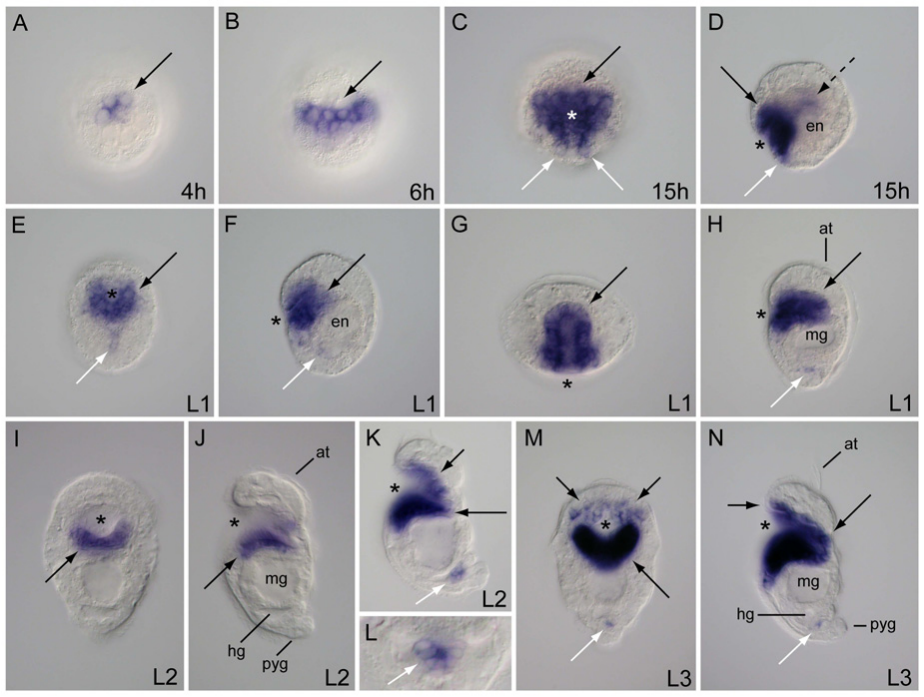
**Developmental expression of *FoxA *in *Chaetopterus***. (A, B) Vegetal views; (C, E, I, M) ventral views with anterior to the top (D, F, H, J, K, N) lateral views with anterior to the top and ventral left; (G) anterior view with ventral down. (A) *FoxA *is expressed in a group of four vegetal cells (arrow) 4 hours after fertilization. (B) Late gastrula-stage embryo 6 hours after fertilization with expression (arrow) on the vegetal plate. (C) Protrochophore larva at 15 hours showing *FoxA *expression in the stomodeum (black arrow) and a pair of ventral posterior cells (white arrows). (D) Same larva as in C, showing *FoxA *expression in the stomodeum (solid black arrow), posterior to the stomodeum (white arrow) and in endoderm (broken black arrow). (E, F) L1 larva at 25 hours with *FoxA *transcription in the stomodeum (black arrow) and subsurface cells (white arrow) along the ventral midline. (G) L1 at 27 hours with *FoxA *expression (black arrow) lining the stomodeal canal. (H) Same larva as in (G), showing expression in the stomodeum (black arrow) and putative hindgut cells (white arrow). (I, J) L2 larva showing positive *FoxA *cells (black arrow) in the posterior side of the stomodeum. (K) Extended color development in L2 larva with expression along the ventral posterior side of the stomodeum (long black arrow), within the stomodeum roof (short black arrow) and in the rectum (white arrow). (L) Posterior dorsal view of larva in K. *FoxA*-positive cells (white arrow) surround the rectal canal. (M, N) L3 larva showing *FoxA *expression along the stomodeum canal (long black arrow), in the stomodeum roof (short black arrows) and the rectal canal (white arrow). Asterisk marks position of the stomodeum. h, hours post fertilization; at, apical tuft; en, endoderm; hg, hindgut; mg, midgut; pyg, pygidium.

The *FoxA *ortholog in *Chaetopterus *is expressed within macromeres on the vegetal plate of the 4 hour late cleavage-stage embryo (Figure 5A). In the 6 hour gastrula, the expression domain includes both surface and subsurface cells on the vegetal face (Figure 5B). In the 15 hour embryo, *Cht-FoxA *is expressed in surface cells surrounding the stomodeum and within stomodeal cells that are internalized during elongation of the stomodeum (Figure 5C, D). *Cht-FoxA *is also expressed in distinct ventral surface cells at the posterior-most edge of the stomodeal expression domain, and internally within the yolky endoderm region, dorsal to the stomodeum (Figure 5C, D). In the early 24 to 25 hour L1 larva, *Cht-FoxA *is expressed in surface cells on the lateral and posterior sides of the stomodeum, and along all internal sides of the stomodeal canal (Figure 5E, F). At the same time, there is expression in a distinct subset of posterior subsurface cells on the ventral midline. As the stomodeum continues to elongate, *Cht-FoxA *expression persists in the epithelial cells of the stomodeum and appears in several cells at the location of the presumptive hindgut (Figure 5G, H). In the functional gut of the L2 larva, expression includes ventral and lateral foregut epithelia, cells along the foregut roof, and pharyngeal diverticula lining dorsal margins of the foregut cavity (Figure 5I-K). Additionally, *Cht-FoxA *is expressed in a ring of cells surrounding the rectal canal (Figure 5K, L). These same foregut and hindgut expression domains are maintained in early-stage L3 larvae of *Chaetopterus *(Figure 5M, N).

**Figure 6:**
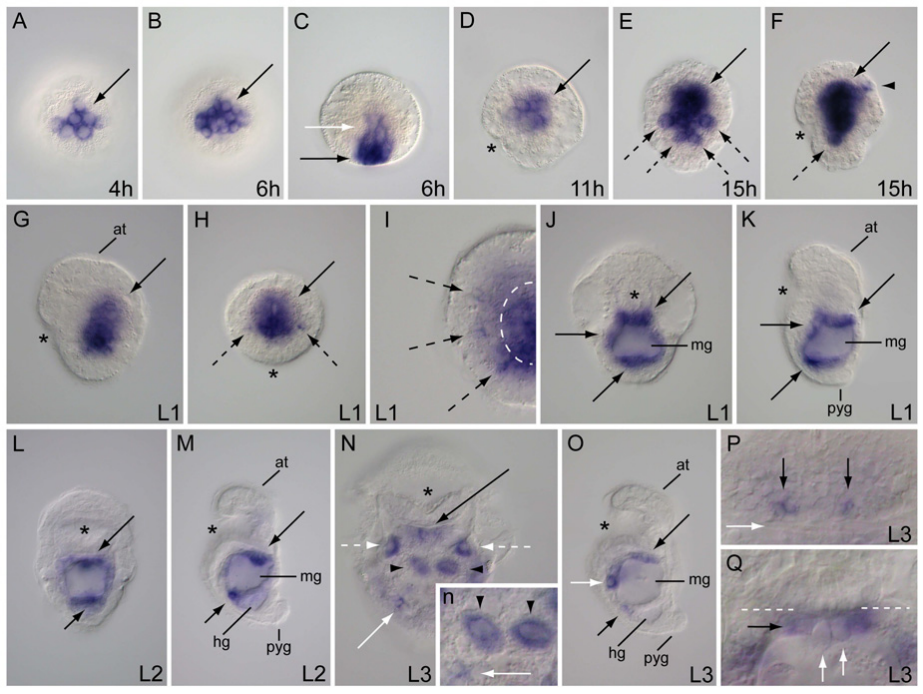
**Developmental expression of *GATA456 *in *Chaetopterus***. (A, B) Vegetal views; (D, F, G, K, M, O) lateral views, anterior to the top, ventral left; (E, I, J, L, N) ventral views, anterior to the top. (A) *GATA456 *expression in vegetal plate cells (arrow). (B) Expression on vegetal surface (arrow) of gastrula embryo. (C) Gastrula in (B) showing expression in surface (black arrow) and subsurface cells (white arrow). (D) Embryo at 11 hours with internal expression (arrow). (E, F) Expression in endoderm (solid black arrows), bilateral cells (broken black arrows) adjacent to endoderm and anterior dorsal cells (arrowhead). (G) Larva at 25 hours showing endodermal expression (arrow). (H) Posterior view of larva in (G) with expression in endoderm (solid black arrow) and outside the endoderm (broken black arrows). (I) Right side of 25-hour larva with *GATA456 *in endoderm (broken white arc) and cells (broken black arrows) flanking endoderm. (J, K) Larva at 27 hours showing expression (arrows) in midgut epithelium. (L, M) Expression is prominent in foregut-midgut (long arrow) and midgut-hindgut (short arrow) junctions. (N) Enlarged ventral view of L3. Expression in foregut-midgut junction (long black arrow), midgut epithelium (solid white arrow), and distinct pairs of lateral (broken white arrows) and medial (black arrowheads) midgut cells. (Inset) magnification of (N) showing expression in medial cells (arrowheads) and smaller surface cells (white arrow) of the midgut. (O) L3 larva with expression in foregut-midgut junction (long black arrow), ventral midgut cell (white arrow), and between midgut and hindgut compartments (short black arrow). (P) Dorsal posterior head region of L3 with *GATA456 *in bilateral cell patches (black arrows) adjacent to pharyngeal diverticula (white arrow). (Q) Dorsal view of foregut-midgut junction (broken white lines) showing expression on midgut side (short black arrow) and in pharyngeal valve cells (white arrows). Asterisk marks position of the stomodeum. h, hours post fertilization; at, apical tuft; hg, hindgut; mg, midgut; pyg, pygidium.

The one *Chaetopterus GATA456 *ortholog we recovered is expressed in both endodermal and mesodermal cells. In the 4 hour embryo, *Cht-GATA456a *appears to be expressed in all four macromeres, and in 4a, 4b and 4c micromeres on the vegetal plate (Figure 6A). At 6 h, *Cht-GATA456a *is expressed in a discrete cluster of vegetal cells both on and below the surface during gastrulation (Figure 6B, C). After 11 hours of development, the expression domain is completely internalized (Figure 6D). There are three domains of expression in the 15 hour embryo. *Cht-GATA456a *is expressed in the central yolk-rich endoderm and in a bilateral pattern in single cells on the lateral posterior sides of the more prominent endoderm domain (Figure 6E). There is also bilateral expression in ectodermal patches on the dorsal anterior surface of the embryo (Figure 6F). In early L1 larval stages, *Cht-GATA456a *is expressed predominantly in the centralized endoderm and in separate lateral patches of cells adjacent to, but outside of the endoderm (Figure 6G-I). Each lateral patch shows expression in 1 to 2 cells, and each patch appears to be separated from the endodermal domain by non-expressing cells (Figure 6I). In mid-stage L1 larvae, *Cht-GATA456a *is expressed in the epithelium surrounding the midgut cavity, with higher expression levels at foregut-midgut and midgut-hindgut junctions (Figure 6J, K). A similar pattern of expression is seen in L2 larvae, although higher transcription levels of *Cht-GATA456a *at the major gut compartment transitions are more pronounced, with lower levels of expression in remaining areas of the midgut epithelia (Figure 6L, M). In the L3 stage, *Cht-GATA456a *is prominently expressed in four comparatively large midgut cells on the ventral anterior face and lateral anterior sides of the midgut (Figure 6N, inset). Across specimens, these cells are consistent in their relative positions, they are distinct in size and morphology from other midgut cells, and one or both of the ventral pair is often found to be binucleate (data not shown). The expression of *Cht-GATA456a *in individual midgut epithelial cells (Figure 6N) and cells at the gut compartment junctions (Figure 6O) are still obvious in L3 larvae. From a dorsal view*, Cht-GATA456a *expression is also apparent in a bilateral pair of cell patches adjacent to the pharyngeal diverticula on the dorsal posterior head region (Figure 6P). At the foregut-midgut transition in L3 larvae, *Cht-GATA456a *is expressed in epithelial cells on the midgut side and in two or more valve cells of the pharynx (Figure 6Q).

### Developmental expression of *Tl-FoxA*, *Tl-GATA456a *and *Tl-GATA456b*

**Figure 7:**
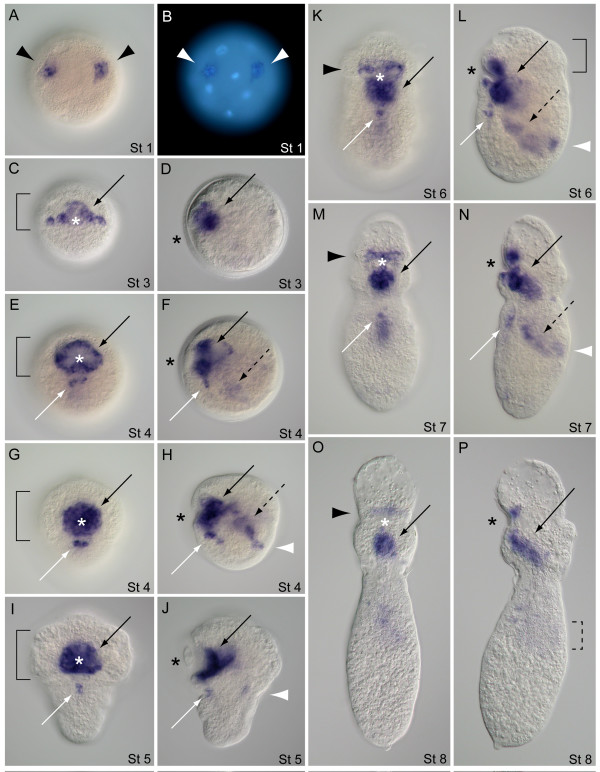
**Developmental expression of *FoxA *in *Themiste lageniformis***. (A, B) Vegetal views; (C, E, G, I, K, M, O) ventral views with anterior up; (D, F, H, J, L, N, P) corresponding lateral views with ventral side to the left, anterior up. (A) Two cells on the anterior vegetal face of the blastula express *FoxA*. (B) Same embryo and orientation as in (A). *FoxA *expression overlaps with Hoechst-labeled nuclei (white arrowheads). (C) Stage 3 expression within and lateral to the invaginating stomodeum (black arrow). (D) Same embryo as in (C), showing subsurface stomodeal expression (black arrow). (E, F) Early stage 4 embryo. *FoxA *expression in the stomodeum (black arrow), subsurface cells on ventral midline (white arrow) and within endoderm (broken black arrow). (G, H) *FoxA *expression in the stomodeum (black arrow) and subsurface cells on the ventral midline (white arrow). (H) Same embryo as in (G) showing expression in endoderm (broken black arrow) and putative hindgut tissue (white arrowhead). (I, J) Stage 5 embryo during transition to the pelagosphera larva. *FoxA *is expressed in the stomodeum (black arrow), ventral ectoderm cells (white arrow), and putative hindgut cells (white arrowhead). (K, L) Posterior elongation of the early larva with expression in the foregut (black arrow), ventral ectoderm (white arrow), midgut (broken black arrow) and presumptive hindgut (white arrowhead). Expression persists on the anterior side of the mouth (black arrowhead). (M, N) Stage 7 pelagosphera with *FoxA *expression in all previous domains. (O, P) Swimming pelagosphera at 3 days. *FoxA *is predominantly expressed in the esophagus (black arrow) and weakly expressed in the presumptive hindgut region (broken bracket). Asterisk marks position of the stomodeum. Solid brackets outline the anterior band of large prototroch cells. Similar arrow types (black, white, broken black) correspond with similar domains of *FoxA *expression.

The transcription of *Tl-FoxA *in *T. lageniformis *was initially detected on the vegetal hemisphere of blastulae. In these stage 1 embryos, *Tl-FoxA *is consistently expressed in a pair of vegetal cells that are separated from each other by a relatively large blastomere that does not express *Tl-FoxA *(Figure 7A, B). Within the cells, *Tl-FoxA *is expressed in cytoplasm surrounding each cell nucleus (Figure 7B). After gastrulation at stage 3, *Tl-FoxA *is expressed on the anterior and lateral sides of the stomodeum in a band of cells spanning the ventral side of the embryo (Figure 7C, D), and there is internal expression that extends dorsally within subsurface cells of the stomodeum (Figure 7D). The lateral band of expression is parallel with and adjacent to the posterior margin of prototroch precursor cells (Figure 7C). In early stage 4 embryos, *Tl-FoxA *is expressed in subsurface cells on all sides of the stomodeum, in a bilaterally symmetric pair of subsurface cell patches posterior to the stomodeal expression, and in a broad expression domain within the interior of the embryo (Figure 7E, F). *Tl-FoxA *is expressed within four regions of late stage 4 embryos: the stomodeum, endoderm, putative proctodeum cells and a bilateral pair of 2 to 3 cells situated below the surface on the posterior ventral side (Figure 7G, H). Expression at the proctodeal region extends into ectoderm cells but is not detectable at the surface. The stomodeum expression is subsurface in cells lining a 'bowl-shaped' depression that is partially enclosed by a band of large prototroch cells. During stage 5, the embryo extends in a posterior direction, forming an overall conical shape, *Tl-FoxA *expression persists in the stomodeum and ventral ectoderm cells, and there is detectable labeling at the site of future hindgut formation (Figure 7I, J). *Tl-FoxA *expression extends internally from the stomodeum in a dorsal-anterior direction where many of the midgut endoderm cells are positioned (Figure 7J). In stage 6, a combination of processes including differential growth, morphogenesis and activity of larval musculature repositions tissues and associated *Tl-FoxA *expression domains along the A/P axis. *Tl-FoxA *is expressed in foregut, midgut and presumptive hindgut cells during this transition from the trochophore-like stage to the early pelagosphera larval form (Figure 7K, L). Expression in ventral ectoderm is still visible, and it is now obvious that this domain is in the ventral nerve cord. All of the above expression domains are maintained in crawling pelagosphera larvae at stage 7, which show the highest levels of expression in foregut and presumptive hindgut tissue (Figure 7M, N). In the swimming pelagosphera, *Tl-FoxA *is predominantly expressed in the foregut, with diminished levels of expression in posterior intestinal tissue. The foregut expression domain includes the main posteriorly descending region and a distinct band of *Tl-FoxA *expression on the anterior side of the mouth that was previously part of a larger expression domain in the stomodeum of pre-larval stages (Figure 7O; see also Figure 7K-M).

**Figure 8:**
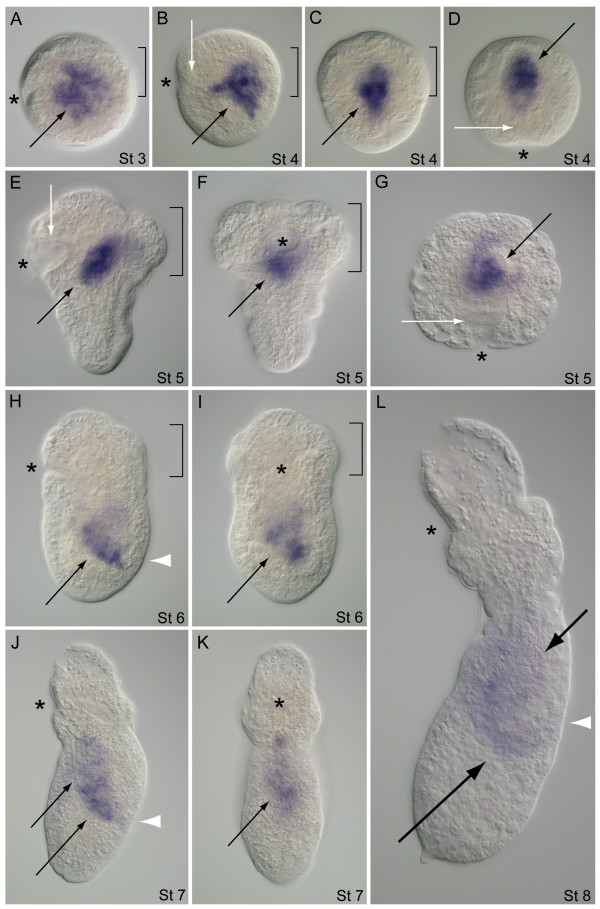
**Developmental expression of the *GATA456a *gene in *Themiste lageniformis***. (A, B, E, H, J, L) Lateral views with ventral side to the left and anterior up; (C) dorsal view with anterior up; (D, G) anterior views with ventral down; (F, I, K) ventral views with anterior up. (A) Post-gastrula showing *GATA456a *expression (black arrow) in the endoderm, which straddles prototrochal (bracket) and posterior posttrochal regions. (B-D) Lateral, dorsal and anterior views, respectively, of the same embryo. *GATA456a *expression (black arrows) is positioned toward the dorsal side of the embryo, internal to the stomodeum (white arrows). (E-G) Lateral, ventral and anterior views, respectively, of the same embryo during the initial stages of posterior elongation. *GATA456a *(black arrows) is expressed internally, surrounded by large prototroch cells (brackets) and dorsal to the stomodeum (white arrows). (H, I) Lateral and ventral views, respectively, during transition to the pelagosphera larva. *GATA456a *is expressed in the posterior trunk (black arrows) and the putative hindgut region (white arrowhead). (J) Lateral view of a crawling-stage pelagosphera. *GATA456a *is expressed in the midgut endoderm (black arrows). White arrowhead marks the position of the future rectum and anus. (K) Ventral view of the same larva as in (J), showing *GATA456a *expression is centralized (black arrow) along the A/P axis. (L) Swimming-stage pelagosphera. The expression of *GATA456a *is expressed in endoderm of the anterior (short black arrow) and descending curve of the intestine (long black arrow). A white arrowhead marks the position of the future anus. Asterisk marks position of the stomodeum. Brackets outline the position of large prototroch cells.

The expression of *Tl-GATA456a *appears to be restricted to endoderm cells and tissues during formation of the midgut. In the stage 3 post-gastrula of *T. lageniformis*, there is a pattern of broad expression that correlates with the centralized yolky region (Figure 8A). At stage 4, the expression domain of *Tl-GATA456a *is more condensed and clearly internal when examined from lateral, dorsal or anterior views (Figure 8B, C). This domain is dorsal to the stomodeum and spans the posterior margin of the large band of prototroch cells. During posterior elongation in stage 5, *Tl-GATA456a *expression remains primarily dorsal to the stomodeum, has a consistent diagonal orientation relative to the dorsal-ventral (D/V) axis, and is in the anterior of the embryo (Figure 8E-G). Once presumptive midgut cells are repositioned to the posterior during stage 6, *Tl-GATA456a *expression is restricted to the trunk region, where the midgut is developing (Figure 8H, I). In the young pelagosphera of stage 7, the expression domain follows the contour of the midgut, which sweeps from ventral anterior to dorsal posterior along the A/P axis (Figure 8J). The midgut expression domain is internal and does not extend to the larval body wall (Figure 8J, K). At stage 8, the overall expression level of *Tl-GATA456a *is lower than in previous stages, and occurs predominantly along both descending and ascending portions of the presumptive intestine (Figure 8L). As with previous stages, there is no expression of *Tl-GATA456a *on the dorsal posterior body wall at the position of the future anus (see Figure 8H, J, L).

**Figure 9:**
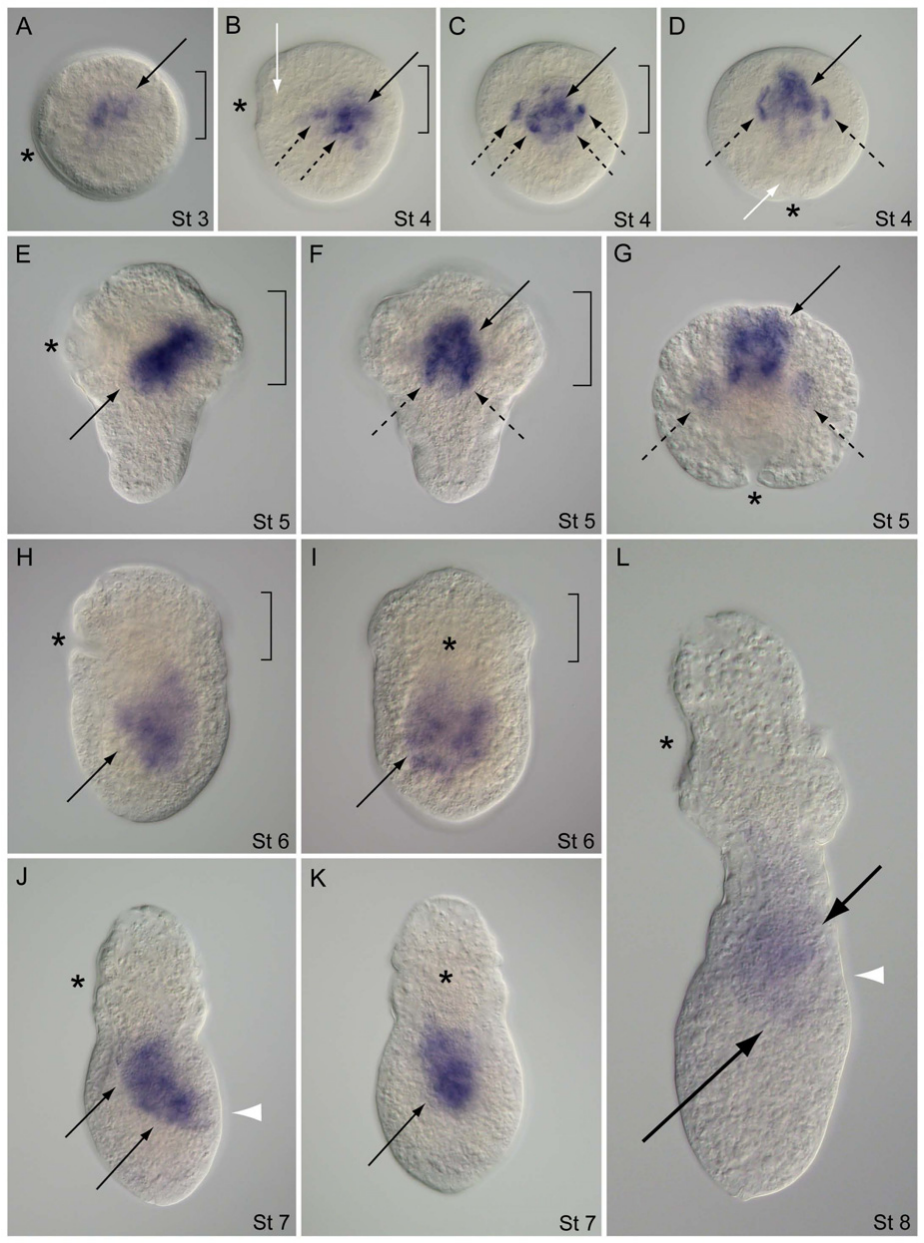
**Developmental expression of the *GATA456b *gene in *Themiste lageniformis***. (A, B, E, H, J, L) Lateral views with ventral side to the left, anterior is up; (C) dorsal view with anterior up; (D, G) anterior views with ventral down; (F, I, K) ventral views with anterior up. (A) Post-gastrula embryo showing *GATA456b *expression localized to a central patch of cells (black arrow) surrounded by the prototroch (bracket). (B-D) Lateral, dorsal and anterior views, respectively, of the same embryo. *GATA456b *is expressed in a patch of endoderm cells (solid black arrows) dorsal to the stomodeum (white arrows) and in discrete cells and cell clusters adjacent to the endoderm (broken black arrows). (E-G) Lateral, dorsal and anterior views, respectively, of the same embryo during the initial stages of posterior elongation. *GATA456b *is expressed in a central anterior domain (solid black arrows), and in cells both outside and flanking each side of the central domain (broken-black arrows). (H, I) Lateral and ventral views of the same specimen during transition to the pelagosphera. *GATA456b *is expressed broadly in the central posterior trunk region (black arrows). (J, K) Lateral and ventral views of the same crawling-stage pelagosphera. *GATA456b *is expressed in the midgut (black arrows). White arrowhead marks the position of the future rectum and anus. (L) Lateral view of a swimming-stage pelagosphera. *GATA456b *is transcribed in regions of the anterior (short black arrow) and descending curve of the intestine (long black arrow). White arrowhead marks position of the future anus. Asterisk marks position of the stomodeum. hour, hours; d, days. Brackets outline the anterior band of large prototroch cells.

The expression pattern of *Tl-GATA456b *overlaps with the endodermal expression observed for *Tp-GATA456a*; however, there is an additional expression domain for *Tl-GATA456b *in the mesoderm. In stage 3 embryos, *Tl-GATA456b *is expressed in several cells positioned dorsal to the stomodeum and within the central yolk-rich interior of the embryo (Figure 9A). During stage 4, two domains of expression are distinguishable: a broader medial group of cells and several distinct lateral patches that are adjacent to the medial expression domain (Figure 9B-D). The lateral patches consist of 2 to 3 cells each, which are separated from each other by non-expressing cells and together form an overall 'V' shape from anterior to posterior (Figure 9C). The cone-shaped stage 5 embryo has a diagonal expression pattern along the D/V axis that is dorsal to the stomodeum (Figure 9E). From a dorsal view, this stage 5 pattern shows two bands of labeled cells that extend posteriorly from the centralized domain (Figure 9F). There is also a bilateral pair of labeled patches outside the central yolky region and separate from the medial domain of *Tl-GATA456b *expression (Figure 9G). During the stage 6 transformation to the early pre-pelagosphera larva, expression is repositioned to the posterior trunk region and is no longer distinguishable as two separate domains (Figure 9H, I). There is a patchy distribution of *GATA456b*-positive cells in the presumptive midgut, before the formation of a midgut epithelium takes place. As with previous stages, expression domains in stage 6 are internal to the ectoderm and do not overlap with any stomodeal cells. Stage 7 pelagospherae show *Tl-GATA456b *expression that extends along the midgut from the posterior end of the foregut to the terminal end of the presumptive hindgut (Figure 9J, K). In the swimming pelagosphera, the expression of *Tl-GATA456b *is reduced but still detectable in the intestine along most of its length, including anterior expression at the esophagus-intestine junction and in posterior cells in the ascending arm of the intestine (Figure 9L).

## Discussion

In this study, we provide novel information on gut development in the sipunculan, *Themiste lageniformis*, which supplements previous work [23]. Using labeling techniques and confocal microscopy, we identified presumptive endoderm and mesoderm cells relative to positional landmarks such as the apical groove, stomodeum, muscle retractors and metatrochal cells. In the trochophore-like stage before elongation, endodermal cells are located adjacent and posterior to brain cells in the anterior of the animal. In lecithotrophic pelagospherae, there is a compact aggregation of stomodeal cells occluding the presumptive esophagus, whereas a symmetric epithelium lines the foregut canal in the planktotrophic larval digestive system of *Chaetopterus*. The posterior end of the larval gut in *Themiste *is initially positioned midway along the dorsal trunk body wall, and both the intestine and larval body elongate posterior to the anal region resulting in the U-shaped gut in adults, a morphology that is considered to be an important adaptation among tubular burrow-dwelling sipunculans [57]. By contrast, the linear gut of polychaetes usually terminates within or immediately adjacent to the pygidium at the worm's posterior end [58], even when the anus is positioned dorsally, such as in the larvae of tubiculous species (such as serpulids and chaetopterids). By comparing gut formation in *Chaetopterus*, *Themiste *and *Capitella teleta *[10], it is clear that a distinct digestive architecture develops in the larvae of each worm, which may correspond with divergent life history modes and feeding mechanisms.

Our results reveal that differences among species in gut architecture are mirrored in the expression patterns of regulatory 'gut genes.' During early development, *FoxA *family orthologs are expressed in the vegetal plate blastomeres of *Chaetopterus*, *Themiste *and *Capitella*; however, *FoxA *expression in the endoderm of *Themiste *is more persistent and extensive. In endodermal cells of *Chaetopterus *and *Capitella*, *FoxA *appears to be downregulated shortly after gastrulation and well before a gut tube is detectable, yet *FoxA *transcripts are expressed in the endoderm of *Themiste *during midgut morphogenesis. This could reflect a longer duration of lecithotrophic development, later onset of feeding, and/or the unique anatomy of sipunculan coiled-helix intestinal systems [56,57,59,60]. Species variation of *FoxA *expression in the foregut is less apparent and may simply be related to the time for completion of a functional gut, which requires 1.5 days, 9 days and 2 to 3 weeks in *Chaetopterus*, *Capitella *and *Themiste*, respectively. In *Chaetopterus*, *FoxA *is expressed in the stomodeum 11 hours after fertilization, well before a distinct foregut epithelium is apparent (27 to 28 hours post-fertilization) and later *FoxA *is expressed at the oral surface in feeding larvae. By contrast, the stomodeum and foregut epithelium are formed after approximately 4 and 5 days, respectively, in the lecithotrophic metatrochophore of *Capitella*, and a distinct foregut epithelium is not even visible in the lecithotrophic pelagosphera of *Themiste*. The *FoxA *gene is also expressed throughout foregut morphogenesis in these two worms, although expression does not extend to the stomodeal surface cells as it does in *Chaetopterus*. Different polychaete species of *Hydroides *(for example, *H. hexagonus*; *H. uncinata*) generate planktonic trochophores with a tripartite gut 20 hours after fertilization [61]http://hdl.handle.net/1912/295 and *H. elegans *has the ability to feed after just 9 hours (B. Nedved, personal communication). Accordingly, Arenas-Mena [52] found high levels of *FoxA *(*He-FoxA1 *and *He-FoxA2*) expression in foregut and endoderm precursors in 3 to 4 hour blastulae of *H. elegans*, although endoderm expression was transient and declined shortly after gastrulation in that polychaete as well. Thus, in the foregut domains of the species mentioned here, *FoxA *is commonly expressed during a broad temporal window from cleavage through larval development, regardless of developmental mode or tempo. In contrast, in the midgut endoderm of *Themiste*, there seems to be a heterochronic shift in the duration of *FoxA *expression relative to expression in the endoderm of polychaetes.

The *FoxA *gene is also expressed in the hindgut of *Chaetopterus*, *Themiste*, *Capitella *[10] and *H. elegans *[52], yet it extends much further internally into presumptive intestinal endoderm in *Themiste*. During stages 6 to 8 in the pelagosphera of *Themiste*, *FoxA *is expressed along the majority of the length of the gut, contrasting with the more restricted, separate foregut and hindgut expression domains of *FoxA *in *Chaetopterus *and *Capitella*. Sipunculans generally have a complex hindgut region in which highly folded, ciliated columnar epithelia and more extensive musculature develop [59]. A *FoxA *ortholog is expressed in both ciliated columnar epithelia and highly muscularized tissues of the pharynx in *Capitella *[10]; however, there is no comparable pharyngeal anatomy in *Themiste *or adult sipunculans [57]. It will be informative to examine *FoxA *expression in the larvae of sipunculan species that have a pharynx, which includes muscularized feeding structures such as the buccal organ and lip gland [29,56,59], to see if there is a significant shift in the amount and extent of *FoxA *expression between anterior and posterior regions in different sipunculans.

In all three species we have examined, *FoxA *expression in the posterior gut is interior to surface ectoderm and may have a role in specifying the transition between hindgut/rectum and anus. In *Drosophila*, there is a 'constellation' of regulatory and signaling genes (*caudal, FoxA/forkhead, brachyenteron, wingless, hedgehog, dpp*) that comprise a regulatory network controlling gastrulation and hindgut development [62-65]. In *Capitella*, there is *FoxA *expression in posterior endoderm and ectoderm, where the hindgut meets cells of the anus. Here, *FoxA *appears to be co-expressed with *caudal *(*cdx*) in this posterior domain [66], and it both precedes and overlaps with posterior expression of *hedgehog *(*hh*), which is adjacent to the expression of *wingless *(*wg/Wnt1*) in the anus [67]. This pattern implies that a gene regulatory network similar to that in *Drosophila *may control hindgut formation in polychaetes. Based on the spatially and temporally conserved patterns of *FoxA *expression in the hindgut and foregut of *Chaetopterus*, *Themiste *and *Capitella*, we hypothesize that *FoxA *acts as a key regulator of anterior and posterior gut formation, and is not restricted to a single germ layer.

Outside the gut, *FoxA *is expressed in a discrete patch of ectodermal cells on the ventral midline in stages 4 to 8 in *Themiste*. Although *FoxA *is also expressed in several ectoderm cells on the ventral midline of *Chaetopterus*, this expression is transient and not detectable in L2 and L3 larvae when nerve fibers appear in this region (N. Meyer, personal communication). In *Themiste*, the patch of *FoxA*-positive cells on the ventral midline persists through 'first metamorphosis' and appears to correspond with serotonergic cells of the ventral nerve cord (VNC) in stages 6 to 7 pelagospherae (N. Meyer, personal communication). It is possible that these patterns reflect temporal differences in VNC specification between planktotrophic (*Chaetopterus*) and lecithotrophic (*Themiste*) development. In *Themiste*, *FoxA *is also expressed in bilaterally symmetric cell clusters along lateral margins of the trunk in stages 6 and 7 (not shown). This and the ectodermal expression at the ventral midline probably correspond to components of the sipunculan nervous system, and represent a co-option of the *FoxA *gene outside its conserved role in gut development.

*GATA *transcription factors are expressed in both the endoderm and mesoderm of *Chaetopterus *and *Themiste*. We isolated a single ortholog in the GATA4/5/6 subclass from *Chaetopterus *and two orthologs from *Themiste*, although additional subclass members may exist. Expression of *GATA *factors in *Themiste *and *Chaetopterus *are consistent with our previous findings for *Capitella *in which we identified three GATA4/5/6 subclass genes [10], each with a distinct expression pattern: *Ct-gataB1 *is expressed in endoderm, *Ct-gataB2 *in endoderm and visceral mesoderm, and *Ct-gataB3 *is restricted to visceral mesoderm. One of the *GATA456 *factors from *Themiste*, *Tl-GATA456a*, appears to be more similar to one of three *GATA456 *factors in *Capitella *than it is to known *GATA *orthologs from any other polychaetes. Our amino acid sequence analysis indicates that these genes (*Tl-GATA456a *and *Ct-gataB1*) are closely related orthologs of each other. Both genes are expressed exclusively in the endoderm, which may indicate a conserved role after gene duplication events within this subclass of *GATA *factors [10,45,53]. In addition, one *GATA *factor each from *Chaetopterus *(*Cht-GATA456a*), *Themiste *(*Tl-GATA456b*) and *Capitella *(*Ct-gataB2*) is simultaneously expressed in both endodermal and mesodermal cell types associated with the midgut, showing conservation of an additional *GATA456 *ortholog in annelid and sipunculan gut development. In the polychaete *Platynereis dumerilii*, Gillis *et al. *[53] identified a single *GATA *factor (*PdGATA456*) whose expression pattern does not include any endoderm (see below), which contrasts with endoderm expression for at least one *GATA456 *factor in *Chaetopterus*, *Themiste *and *Capitella*.

*GATA456 *expression in mesoderm varies between polychaete annelids. During early development, a stereotypic spiral cleavage program is common among annelids, sipunculans and other lophotrochozoans (for example, echiurans, molluscs, nemerteans, polyclad turbellarians), in which the 4 d cell is termed the 'mesentoblast,' and typically gives rise to adult mesoderm [68-70]. The single *GATA *factor (*PdGATA456*) identified in *Platynereis *[53] is expressed in cells of the paired mesodermal bands, "most likely in descendants of the mesodermal progenitor cells 4d^1 ^and 4d^2^." An important contrast between their study and ours is that in *Chaetopterus*, *Cht-GATA456a *is expressed in seven vegetal-plate blastomeres, which appear to include all fourth quartet micromeres except 4 d, the progenitor of 4d^1 ^and 4d^2^. A similar pattern was found in *Capitella *where *Ct-gataB1 *expression is reduced in 3 D just before the birth of 4 d [10]. Thus, in *Chaetopterus *and *Capitella*, transcription of one *GATA*456 factor seems to be segregated away from the 4 d lineage, indicating that there is species-specific variation of *GATA456 *expression in annelid mesodermal precursors during early development. At later stages, the expression of *PdGATA456 *is detectable throughout the entire mesodermal band [53], yet the expression of a *GATA456 *ortholog is clearly discontinuous among presumptive mesoderm cells flanking the endoderm in *Chaetopterus*, *Themiste *and *Capitella*, which indicates expression within a mesoderm sublineage. Moreover, Gillis *et al. *[53] reported that *PdGATA456 *expression is associated with the segmentally repeated chaetal sacs. The segmental expression of *GATA456 *is not associated with chaetal development in *Capitella *or in epidermal development in the achaetous non-segmented sipunculan, *Themiste*; however, *GATA456 *expression has not been examined during chaetal development in *Chaetopterus*.

We observed several other differences between species in the expression patterns of *FoxA *and *GATA456*. Neither *FoxA *nor *Cht-GATA456a *is expressed in the hindgut of *Chaetopterus*, although there was *GATA456 *expression in the corresponding regions of *Themiste *and *Capitella*. There may be another *GATA *ortholog or even a different regulatory gene in *Chaetopterus *that is expressed in this hindgut tissue. Additionally, because there are no discrete compartments in the larval gut of *Themiste*, *GATA456 *expression does not correlate with distinct gut compartment boundaries, whereas the segregation of *FoxA *and *GATA456 *expression into distinct gut compartments is definitive in *Chaetopterus*. The *GATA456 *expression pattern in the gut of *Themiste *may instead represent the embryonic origin of this tissue (that is, in endodermal precursors). Finally, in *Chaetopterus*, *Cht-GATA456a *appears to be expressed in specialized cells in the midgut (endoderm) and head ectoderm (neural) that are not directly comparable to any patterns of *GATA456 *expression in *Themiste*, *Capitella *or *Platynereis*. This represents a possible co-option of the *Cht-GATA456a *gene in cell types that are present in *Chaetopterus *but not in the other animals. Collectively, it appears that particular developmental roles for members of the *GATA456 *subclass have been partitioned among species and between germ layers during the diversification of annelids.

## Conclusions

**Figure 10:**
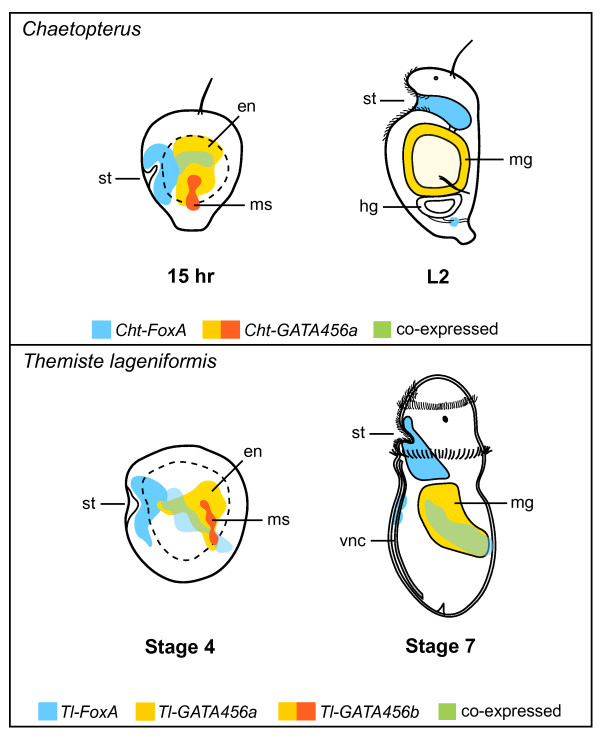
***FoxA *and *GATA456 *are regionally expressed along the alimentary canal in *Chaetopterus *and *Themiste lageniformis***. Lateral views of (left) post-gastrula and (right) larval stages showing a summary of expression in presumptive and definitive gut regions. Colors correspond to individual *FoxA *and *GATA456 *expression patterns and overlapping domains where both transcription factors are co-expressed. One *GATA456 *gene in each species is simultaneously expressed in endoderm and mesoderm. Blue, *FoxA*; yellow and yellow + red, *GATA456*; dotted line, yolk-rich interior. en, endoderm; hg, hindgut; mg, midgut; ms, mesoderm; st, stomodeum; vnc, ventral nerve cord.

Although we observed several species-specific differences, an overall comparison of *FoxA *and *GATA456 *transcription along the alimentary canal reveals a conserved trend of regionalized expression in these two species (Figure 10). This trend is even more apparent when also considering the expression patterns previously described in *Capitella*. *FoxA *appears to be involved in patterning anterior and posterior regions of the larval digestive system, regardless of the rate of development or whether the developmental mode is planktotrophic or lecithotrophic. Orthologs of the *GATA456 *subclass also show conserved expression localized to the midgut and a mesoderm sublineage. Both *FoxA *and *GATA456 *transcripts are largely restricted to tissues of the gut tube, unlike other gut-related genes, which are expressed in additional domains external to the digestive system [3]. Together, the expression of *FoxA *and *GATA456 *span most of the length of the gut tube in *Chaetopterus*, *Themiste *and *Capitella*. Furthermore, *FoxA *and *GATA456 *show spatial and temporal overlap at the ectodermal-endodermal transitions along the gut tube, and there is almost complete overlap in the midgut of a sipunculan. This would suggest the possibility of transcriptional cooperation or 'genetic potentiation,' which has been characterized in detail for the HNF3/FoxA-GATA partnership in mouse embryonic endoderm [3,5].

It is likely that both transcription factors are near the top of gut-specific gene regulatory networks in spiralians. Orthologs of *FoxA *and *GATA *factors are integral components of endomesoderm networks in a nematode [46,71] and sea urchin [50,55] and required for digestive tract development in fly [72,73] and mouse embryos [74,75]. If we also consider the expression patterns in embryos as diverse as a hemichordate [76], mollusc [77,78] or an anthozoan cnidarian [79], there is evidence of ancient conservation or 'deep homology' [80] for the roles of *FoxA *and *GATA456 *genes in patterning metazoan intestinal systems. Regionalized expression of *FoxA *and *GATA456 *orthologs along the gut tube of annelid lophotrochozoans, taken together with conserved expression patterns in ecdysozoan and deuterostome animals, implies that a through gut may have been present during the earliest stages of bilaterian evolution, even in the bilaterian ancestor itself. Although it is clear that lophotrochozoans use a common set of core gut patterning components, our results reveal significant molecular differences between species and could indicate modifications leading to the evolution of diverse intestinal systems. Investigations of genes further downstream may uncover more dramatic species-specific differences in the process of gut-related tissue specification or cell differentiation.

## Methods

### Animal collection and handling

Adult polychaete worms of *Chaetopterus variopedatus *Cuvier 1827, sensu Enders (1909) were collected near Woods Hole, MA, USA by the Marine Resources Center at Marine Biological Laboratory (MBL). M. E. Petersen (personal communication) examined the material from mud bottoms in the Woods Hole region and has suggested that it probably is not *C. variopedatus*, but a new, undescribed species. Mature worms were maintained in their tubes in flowing seawater for 1 to 2 weeks. Gamete collection, fertilization and the handling of embryos and larvae were conducted according to published protocols [15].

Adult sipunculan worms of *Themiste lageniformis *were extracted from coral rubble found in the shallow subtidal region along the south shore of Oahu, Hawaii. Coral 'boulders' were maintained in flowing seawater tables at Kewalo Marine Laboratory, University of Hawaii at Manoa for up to 1 to 2 months and broken open to collect worms. Mature specimens were placed into glass finger bowls containing filtered seawater (FSW; 22 μm filter) for 1 to 2 hours, followed by dissection of coelomic contents into plastic petri dishes coated in a gelatin solution (1.0% gelatin; 0.37% formaldehyde; dH_2_O). Mature oocytes were isolated from other coelomic materials by washing in FSW. Embryonic development of *T. lageniformis *was activated by treatment with 1.0 mM cAMP (N, 2'-O-dibutyryladenosine 3': 5- cyclic monophosphate; Sigma, St. Louis, MO, USA) in coated petri dishes containing oocytes and FSW. cAMP stock solution was prepared at 0.5 M in dimethylformamide (DMF). cAMP treatments were terminated after 1.5 hours by several FSW exchanges. Developing embryos and larvae were maintained at room temperature (RT) with daily exchanges of FSW containing antibiotics (0.6 mg/ml penicillin; 0.5 mg/ml streptomycin). A detailed protocol is available upon request.

### Cloning of *FoxA *and *GATA456 *genes

Total RNA was collected from *Chaetopterus *oocytes, embryos at 3 to 9 hours post-fertilization, and mixed larval stages. 5' and 3' cDNA templates were synthesized using rapid amplification of cDNA ends (RACE (SMART RACE system; Clontech, Mountainview, CA, USA). DNA fragments of *Chaetopterus FoxA *and *GATA456 *were recovered from the cDNA template by degenerate PCR. A 249 bp fragment of the *Cht-FoxA *winged-helix domain was isolated with the following nested primer set: A1Fout: 5'-AAR CCN CCN TAY WSN TAY AT -3' and A2Rout: 5'-TAR CAN CCR TTY TCR AAC AT-3', followed by A2Fin: 5'-TAY ATH WSN YTN ATH CAN ATG -3' and A1Rin: 5'-CCR TTY TCR AAC ATR TTN CC -3'. A 348 bp fragment of the *Cht-GATA456 *C_4 _zinc-finger domain was isolated using the following semi-nested primer set: GTF1out: 5'-GAR TGY GTN AAY TGY GGN GC -3' and GTR1out: 5'-GGY TTN CKY TTN CKN GTY GT -3', followed by GTF2in: 5'-GGN CAY TAY YTN TGY AAY GC -3' and GTR1out. Gene-specific RACE primers were designed (MacVector Inc., Cary, NC, USA), synthesized (IDT, San Diego, CA, USA) and used (Advantage^® ^2 PCR Kit; Clontech). RACE fragments of 1389 bp (3') and 737 bp (5') were recovered for *Cht-FoxA *and fragments of 1038 bp (3') and ~ 1490 bp (5') were recovered for *Cht-GATA456a*.

Total RNA was collected from *T. lageniformis *oocytes and from assorted embryonic and larval stages from the first 3 days of development. 5' and 3' RACE-Ready cDNA templates were prepared as described for *Chaetopterus*. A degenerate DNA fragment of 226 bp from the *Tl-FoxA *winged-helix domain was isolated using the following semi-nested primer set: A1Fout and A2Rout, followed by A1Fout and A3Rin: 5'-CCA RAA NSW NCC YTT NCC NGG -3'. A 339 bp degenerate fragment of *Tl-GATA456 *was isolated with the primer set: GTF1out and GTR1out. Gene-specific RACE primers were designed and used as previously described. RACE fragments of 1569 bp (3') and 779 bp (5') were recovered for *Tl-FoxA*. Two distinct *GATA456 *genes were recovered from *T. lageniformis *using one set of 5' and 3' RACE primers designed from the same conserved, C_4 _zinc-finger domain: RACE fragments of 1485 bp (3') and 1102 bp (5') for *Tl-GATA456a *and 1068 bp (3') and 881 bp (5') for *Tl-GATA456b *were recovered. Additional gene-specific primers were then used to confirm the identity of each distinct *Tl-GATA *factor by amplification of sequences connecting each of the individual *Tl-GATA456a *and *Tl-GATA456b *RACE fragments. Degenerate and RACE gene fragments obtained from both species were subcloned into pGEM-T Easy vectors (Promega, Madison, WIm USA) and sequenced at Macrogen Inc. (Seoul, South Korea).

### Gene orthology analyses

Sequence data for all recovered gene fragments from *Chaetopterus *and *T. lageniformis *were examined for similarity to public database protein sequences using the blastx program of NCBI http://www.ncbi.nlm.nih.gov. Sequence data for DNA-binding domains of the Fox winged-helix and GATA C_4 _zinc-finger proteins from a subset of additional organisms were obtained from the NCBI Protein database (Entrez Protein). All amino acid sequences were aligned using the ClustalW algorithm in MacVector (version 10.6), and manually adjusted for alignment errors.

Phylogenetic analyses of selected Fox winged-helix and GATA zinc-finger domains were performed with MrBayes (version 3.1.2) [81]. The Fox protein analysis used the WAG model of amino acid replacement for 3,000,000 generations sampled every 100 generations, with four chains and four independent runs. A 50% majority-rule consensus tree was generated from the last 90,004 trees. The GATA protein analysis used the JTT model of amino acid replacement for 3,000,000 generations sampled every 100 generations, with four chains and four independent runs. A 50% majority-rule consensus tree was generated from the last 80,004 trees. Gamma shape parameters and the proportion of invariable sites were uniformly distributed for each analysis. The resulting consensus trees were viewed with FigTree (version 1.1.2; http://tree.bio.ed.ac.uk/) and edited in Adobe illustrator CS3.

### Gene accession numbers

Nexus files of the Fox and GATA amino acid alignments are available upon request.

Amino acid sequences used in the alignments are available with the GenBank accession numbers from NCBI http://www.ncbi.nlm.nih.gov/[82] or the identification numbers of predicted proteins from JGI http://www.jgi.doe.gov/[83]:

Fox transcription factors:

Pvfkh [GenBank:AJ507424]; LgFoxA [JGI protein ID:54814]; TlFoxA [GenBank:GU357822]; CtFoxA [GenBank:EF651787]; SpFoxA [GenBank:DQ459376]; PfHNF3 [GenBank:AB023019]; ChtFoxA [GenBank:GU357819]; Pdfkh [GenBank:AM114771]; HrFoxA [JGI protein ID:137725]; Dmfkh [GenBank:J03177]; Bmsgf1 [GenBank:NM_001043864];

Tcfkh [GenBank:NM_001039414]; Atfkh [GenBank:BAC24088]; DrFoxA1 [GenBank:AAH65668]; MmFoxA1 [GenBank:AAH96524]; DrFoxA2 [GenBank:BC086703]; MmFoxA2 [GenBank:NM_010446]; CsHNF3 [GenBank:AB04958]; BfHNF3-1 [GenBank:X96519]; Nvfkh [GenBank:AY457634]; LgFoxB [JGI protein ID:75592]; Pdest [GenBank:CT033411]; Spfkh1 [GenBank:NP_999797]; XlFoxB [GenBank:AJ487619]; BfFoxB [GenBank:AJ506162]; CtFoxB [JGI protein ID:225366]; NvfkhB [GenBank:DQ17368]; SpFoxABlk [GenBank:XM_001186527]; CtFoxAB [JGI protein ID:131123]; CtFoxC [JGI protein ID:199610]; SpFoxC [GenBank:XM_001183474]; Dmcroc [GenBank:NM_079478]; BfFoxC [GenBank:CAH69694]; SpFoxG [GenBank:DQ286739].

GATA transcription factors:

CeELT1 [GenBank:BAE06473]; CbELT1 [GenBank:XM_002633918]; DmGRAIN [GenBank:NP_731211]; TcGRAIN [GenBank:NM_001164788]; SpGATAc [GenBank:AAC62960]; CiGATAb [GenBank:BAE06473]; DrGATA1 [GenBank:NM_131234]; MmGATA1 [GenBank:NP_032115]; DrGATA2 [GenBank:NM_131233]; MmGATA2 [GenBank:BC107009]; DrGATA3 [GenBank:BC162401]; MmGATA3 [GenBank:NM_008091]; LgGATAc [JGI protein ID:160340]; ChtGATA123a [GenBank:GU357821]; PdGATA123 [GenBank:ABK32792]; CtGATAa1[GenBank:EF651791]; NvGATA [GenBank:AY465174]; CtGATAb2 [GenBank:EF651789]; PdGATA456 [GenBank:ABK32792]; SpGATAe [GenBank:NM_001005725]; ChtGATA456a [GenBank:GU357820]; TlGATA456b [GenBank:GU357824]; DmPANNIER [GenBank:NM_057337]; AgPANNIER [GenBank:AF395080]; AaGatad [GenBank:AY745809]; DmSERPENT [GenBank:NP_732098]; AmSERPENT [GenBank:XM_001121273]; AaGata4 [GenBank:EAT37835]; TcGATA [GenBank:XM_968040]; DrGATA4 [GenBank:NM_131236]; MmGATA4 [GenBank:AF179424]; DrGATA5 [GenBank:BC116537]; MmGATA5 [GenBank:AK142213]; DrGATA6 [GenBank:BC067710]; MmGATA6 [GenBank:NM010258]; LgGATAb [JGI protein ID:167550]; LgGATAa [JGI protein ID:129788]; CtGATAb3 [GenBank:EF651790]; CtGATAb1 [GenBank:EF651788]; TlGATA456a [GenBank:GU357823].

### Whole mount *in situ *hybridization

*Chaetopterus *embryos were pretreated with a 1:1 mixture of 1.0 M sucrose and 0.25 M sodium citrate (Sigma, St. Louis, MO, USA) for 1 to 2 minutes, washed in FSW to terminate pretreatment, and fixed with 3.7% formaldehyde in FSW overnight at 4°C. *Chaetopterus *larvae were relaxed in a 1:1 mixture of 0.37 M MgCl_2 _in FSW for 10 to 15 minutes and fixed in 3.7% formaldehyde in FSW overnight at 4°C.

*T. lageniformis *stage 3 to 4 embryos were pretreated with a 1:1 mixture of 100 mM sucrose:0.25 M Na citrate for 2 to 3 minutes, washed in FSW and fixed in 4% paraformaldehyde in FSW. Stage 5 pre-larvae were anesthetized with 1:1 0.37 M MgCl_2 _in FSW for 30 to 40 minutes, followed by a 'slow-fix' with drop-wise (5 to 10 μl) additions of 4% paraformaldehyde every 10 minutes until no movement was detected, then fixation with 4% paraformaldehyde in FSW. Stage 6 to 8 pelagosphera larvae were anesthetized with a 1:1:2 mixture of 0.37 M MgCl2: 0.25% bupivicaine hydrochloride (BH; Sigma) in FSW for 1 hour, followed by fixation with 4% paraformaldehyde in FSW. All fixations were carried out overnight at 4°C.

Specimens of both species were washed out of fixative into phosphate-buffered saline (PBS), dehydrated stepwise into 100% methanol, and stored at -20°C. Before hybridization, specimens were rehydrated stepwise from methanol into PBS with 0.1% Tween-20 detergent (PTw). Whole mount *in situ *hybridization was performed according to published protocols [18,67] with several modifications: proteinase-K digestion was terminated after 2 minutes (*Chaetopterus*) or 15 minutes (*T. lageniformis*); acetic anhydride treatments were 6.0 μl/ml and 12.0 μl/ml triethanolamine (*Chaetopterus*) or 4.0 μl/ml and 8.0 μl/ml triethanolamine (*T. lageniformis*); pre-hybridization consisted of 50% hybridization buffer for 10 minutes followed by 100% hybridization buffer overnight (*Chaetopterus*) or 100% hybridization buffer overnight (*T. lageniformis*). For both species, *in situ *hybridization was conducted at 65°C for 72 hours, with the following color development reaction: [4.4 μl nitroblue tetrazolium (NBT) 75 mg/ml: 3.3 μl 5-bromo-4-chloro-3-indolyl phosphate (BCIP) 50 mg/ml]/ml alkaline phosphatase (AP) buffer. A detailed *in situ *hybridization protocol is available upon request.

A 5' riboprobe of 737 bp was synthesized for *Cht-FoxA*; a 5' riboprobe of approximately 1490 bp was synthesized for *Cht-GATA456a *(each used at 0.5 ng/μl). A 3' riboprobe of 1569 bp was synthesized for *Tl-FoxA *(used at 1.0 ng/μl); and two 5' riboprobes of 1102 and 881 bp were synthesized for *Tl-GATA456a *and *Tl-GATA456b*, respectively (each used at 3.0 ng/μl). All of the antisense digoxigenin-labeled riboprobes (dig-11-UTP) were synthesized with a T7 MEGAscript kit (Ambion Inc, Austin, TX, USA) and detected by chromogenic staining (NBT/BCIP) of an anti-digoxigenin antibody-alkaline phosphatase conjugate (Roche, Indianapolis, IN, USA). Color reactions were terminated in PTw, followed by a graded dilution series of hybridization buffer/PTw washes. Specimens were equilibrated and stored in glycerol (80% glycerol, 10% 10X PBS, 10% diH20) and mounted on coated slides (Rainex^®^; Sopus Products, Houston, TX, USA). Microscopic analyses were performed on a compound light microscope (Axioskop 2; Carl Zeiss, Inc. Thornwood, NY, USA) with DIC optics. Digital micrographs of riboprobe-labeled and non-labeled, fixed animals were captured with a stem-mounted, 4.0 megapixel digital camera (Coolpix 4500; Nikon Inc., Melville, NY, USA).

### Confocal imaging and analysis

*T. lageniformis *embryonic and larval stages were pretreated as previously described, fixed for 1.0 hour in 4% paraformaldehyde in FSW at RT, and washed in phosphate-buffered saline with 0.1% Triton X-100 (PBT). Embryos at stages 3 to 5 were blocked in PBS-T with 10% heat-inactivated goat serum (Sigma) for 1 hour at RT, followed by consecutive overnight treatments at 4°C with primary mouse anti-histone antibody (1:250 dilution; F152.C25.WJJ; Millipore, Billerica, MA, USA) and secondary goat anti-mouse rhodamine antibody (1:200 dilution; Molecular Probes, Eugene, OR, USA) in blocking buffer. Secondary antibody was co-incubated with 1:200 BODIPY FL-phallacidin (Molecular Probes). Labeled embryos at stages 3 to 4 were rinsed in PBS and mounted in SlowFade Gold (Molecular Probes). Pelagosphera larvae at stages 6 to 8 were treated with RNase A at 1.0 mg/ml PBS-T for 1 hr at 37°C, washed in PBS-T, and then labeled with propidium iodide (Sigma)] 5 μg/ml in PBS-T and 1:200 BODIPY FL-phallacidin for 2 hours at RT. Stage 5 pre-larvae and pelagosphera larvae were washed in PBS, dehydrated stepwise in isopropanol, cleared by emersion in 2:1 benzyl benzoate:benzyl alcohol, mounted on non-coated glass slides and sealed with clear nail polish. Visualization and imaging of all stages were performed using a confocal microscope (Axioplan 2 LSM510; Zeiss).

## Competing interests

The authors declare that they have no competing interests.

## Authors' contributions

MJB performed molecular cloning, sequence analyses, gene expression protocols, microscopic imaging and writing of the manuscript. ECS provided critical analyses of the data, and helped to design the study and write the manuscript. Both authors read and approved the final manuscript.
